# Strategy and Motivation, Rather Than Fatigue, Drive Age‐Related Differences in Sustained Attention Performance: Evidence for Decoupled Beta‐Band Oscillations

**DOI:** 10.1111/ejn.70402

**Published:** 2026-01-21

**Authors:** Simon Hanzal, Gemma Learmonth, Gregor Thut, Monika Harvey

**Affiliations:** ^1^ School of Psychology and Neuroscience University of Glasgow Glasgow UK; ^2^ Division of Psychology University of Stirling Stirling UK; ^3^ The Brain and Cognition Research Centre (Cerveau et Cognition, Cerco), CNRS UMR5549 and University of Toulouse Toulouse France

**Keywords:** age strategy, beta oscillation, EEG frequency band analysis, fatigue, motivation, visual attention

## Abstract

Reduced vigilance can be captured as attentional lapses in sustained attention tasks, but just how these lapses relate to task‐induced fatigue and motivation to maintain optimal performance across the age span is unclear. We induced fatigue in 18 young (mean age = 22.6 years) and 16 older participants (mean age = 66.5) using the Sustained Attention to Response Task while simultaneously recording electroencephalography (EEG). In the final block, we manipulated motivation levels in half of the participants by offering a financial incentive for best overall performance. We found that the young and older adults differed markedly in their response strategies from the outset (adopting distinct speed‐accuracy trade‐off strategies) with faster/more erroneous responses in the young adults and slower/more accurate responses in the older participants that remained stable over the experiment, while subjective fatigue increased irrespective of group. Poststimulus EEG activity showed two distinguishable beta signatures: a fronto‐central topography as a marker of the age‐specific response strategy and a fronto‐parietal signal modulated by motivation per se. We speculate that these two signatures contribute to offsetting performance declines over time. Finally, although subjective fatigue or mind‐wandering scores and prestimulus alpha power increased with time‐on‐task, we did not identify a correlation between these measures. Hence, strategy and motivation more than fatigue were associated with performance differences across age in a sustained attention task, reflected in decoupled beta signatures.

## Graphical abstract

Markers of vigilance during sustained attention in both young and older participants: stable performance strategies, increase in subjective fatigue, increase in task‐related beta oscillations and distinct brain‐behaviour links.

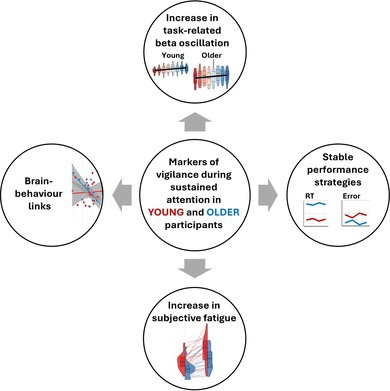

**Abbreviations**
CRT: cathode ray tube
EEG: electroencephalograph
ICA: independent component analysis
MoCA: Montreal Cognitive Assessment
MW‐S: Mind‐Wandering—Spontaneous Scale
SART: sustained attention to response task
UK: United Kingdom
VAS‐F: Visual Analogue Scale for Fatigue

## 1 Introduction

Sustaining attention requires a constant, self‐directed maintenance of vigilance (Robertson and Garavan 2004) both across various daily activities (Massar et al. 2018; Roach et al. 2012; Walker and Trick 2018) and bespoke experimental tasks (Head and Helton 2012; Reteig et al. 2019). Continuously engaging in sustained attention tasks (SART) can result in changes in performance (Reteig et al. 2019; Stoll et al. 2016) that have been characterised as the *vigilance decrement* (Oken et al. 2006; Robertson and Garavan 2004). However, it is well documented that there is large interindividual variability in sustained attention performance over time across the population (Hanzal et al. 2024; Vallesi et al. 2021). Accordingly, the onset of a vigilance decrement can vary across experiments and studied populations. Some studies point to a continued ability of participants to concentrate and maintain focus during SARTs (Lara et al. 2014; Nakagawa et al. 2013), while others suggest impairments in accuracy and reaction times with increasing time‐on‐task (Pershin et al. 2023; Smit et al. 2004; van Schie et al. 2012). With respect to differences in studied populations, different age groups in particular tend to adopt either an accuracy (Dang et al. 2018; Reteig et al. 2019) or speed‐based (Lara et al. 2014; Statsenko et al. 2020) strategy. These strategies are then prone to change during the task, for example, switching from an emphasis on achieving high accuracy to responding faster (van Schie et al. 2012). These changes could reflect age differences in either motivational levels (Carr et al. 2022; Ryan and Campbell 2021) or levels of fatigue (Gilsoul et al. 2022; Yoon et al. 2023). Here, we aimed to study to what extent differences in strategy, motivation and/or fatigue determine performance in a SART across two age groups (young versus older participants) by tracking behavioural measures and EEG correlates over time.

As to motivation, this has been suggested to be of key influence in the reappraisal of task strategies (Earle et al. 2015; Gilsoul et al. 2022), which can lead, through the self‐regulatory mechanism of attentional effort (Sarter et al. 2006; Stoll et al. 2016), to improved behavioural measures in SARTs (Oken et al. 2006). This was tested in young adults by Reteig et al. (2019), who reactivated participants' motivation after 60 min spent on a SART, by offering an additional monetary reward if they managed to outperform 65% of the other participants in the final part of the experiment. Although Reteig et al. (2019) found that the motivational manipulation restored the vigilance decline to some extent, this was not reflected in the tested EEG measures of attentional control, except for variability in a neural theta‐response. Hence, a neural link between the effect of motivation and vigilance change remains tentative (see also Awh et al. 2012). In the present study, we sought to re‐examine the influence of motivation on vigilance decrements by manipulating motivation in the course of sustained attention performance while recording its EEG markers, similarly to Reteig et al. (2019) but comparing across age groups. We anticipated motivated as compared to unmotivated participants to show different performance and oscillatory patterns as a function of age group. Specifically, as argued and then demonstrated in Hanzal et al. (2025), we expected younger participants to respond differently to a motivation manipulation (e.g., with increased accuracy) than older adults, given that older adults have been shown to be less prone to behaviour shifts due to high intrinsic (ceiling) motivation levels stemming from their beliefs that participation in research has a benefit to society and a positive contribution per se (Ryan and Campbell 2021). However, the oscillatory markers of motivational differences have yet to be investigated and will be studied here.

Alongside motivational effects, differences in state fatigue are expected to influence performance declines in SARTs. The exact mechanisms driving such fatigue are still unclear (Kuppuswamy 2022), but are likely caused by either a depletion of cognitive resources (Jacquet et al. 2021; Krigolson et al. 2021) or by monotonous tasks leading to a disengagement of the sustained attention networks (Richard et al. 2018). Notably, previous work highlights that the effect of fatigue on vigilance decrement may be decoupled from the effect of motivation (Gergelyfi et al. 2015; Lorist et al. 2009) and hence taking fatigue into account in the study of neural markers of vigilance change, in addition to motivational factors, is relevant. Regarding the effects of age, although some studies have found higher levels of situational fatigue in older adults (Hess and Knight 2021), others have instead reported a lack of time‐on‐task fatigue in older compared to younger adults (Gilsoul et al. 2022). Two recent behavioural studies investigating sustained attention by Hanzal et al. (2024, 2025) failed to find age‐specific fatigue effects despite the latter experiment demonstrating greater motivational effects (on accuracy) in a younger compared to an older age group, further suggesting a possible decoupling of the two effects.

To track vigilance over time, we here used the Sustained Attention to Response Task (SART) (Robertson et al. 1997; Weinstein 2018) in which failures in sustained attention manifest in commission errors (false positives) arising from erroneously responding to infrequent no‐go stimuli. This contrasts with Reteig et al. (2019), who used a sustained attention‐style task where the goal was to detect rare targets in an oddball paradigm (and where the typical errors were omissions). Performance changes in the SART are influenced by response strategy and age (Dang et al. 2018; Lara et al. 2014; Wilson et al. 2016), can capture lapses into a less attentive state (Wilson et al. 2016), as well as task monotony (Head and Helton 2012), so the mapped effects extend well beyond the task itself (Smit et al. 2004). Prolonged versions of the SART therefore represent a suitable method for exploring the relationship between vigilance decrements and potential differences in age strategy and its links to motivation and fatigue.

Furthermore, we designed our EEG analysis approach to cover signals from the theta to the beta band. This was because EEG research has broadly characterised activity of the vigilance network as occurring in the alpha frequency band (Clayton et al. 2015; Sadaghiani and Kleinschmidt 2016), mapping onto frontoparietal networks (Clayton et al. 2015; Corbetta and Shulman 2011). In line with this view, alpha activity at baseline or a prestimulus interval has been observed to change over time on task (Benwell et al. 2019; G. Li et al. 2020; Macdonald et al. 2011). Previous literature further suggests patterns of change in alpha oscillations connected to a decline in performance in sustained attention (Braun et al. 2015; Nan et al. 2024; Oken et al. 2006). There is some evidence of a connection between posterior alpha increase and task‐induced fatigue (Barwick et al. 2012; Jacquet et al. 2021; Tanaka et al. 2012), as well as general fatigue levels (Maciejewska and Moczarska 2023), while others maintain the link is uncertain (Huycke et al. 2021; Talukdar et al. 2019). Oscillatory activity related to attentional control may also extend beyond the alpha band: Changes in beta‐band activity have been associated with the regulation of attentional effort during time on task, in addition to motor preparation (characterised as beta rebound) (Z. Li et al. 2022; Liu et al. 2010; Stoll et al. 2016). In addition, Reteig et al. (2019) found that a motivational manipulation was linked to changes in the variability of the neural theta response. Thus, analyses of oscillatory signals across the theta, alpha and beta bands should contribute to a better understanding of the neural processes underlying vigilance changes and clarify their contribution to age‐related strategy, with links to motivation and fatigue.

In brief, in this preregistered study, we aimed to investigate the behavioural and EEG measures of task strategy and fatigue in young and older participants as a function of time‐on‐task during prolonged (45 min) SART performance. We also induced higher levels of motivation in half of the participants during the final experimental block to investigate whether motivation could improve performance and change EEG markers of fatigue and further tested for differences between age groups. Our results reveal two dissociated frequency‐specific EEG signatures: one for age‐specific response strategies (poststimulus fronto‐central beta) and one for motivation per se (poststimulus fronto‐parietal beta), on the backdrop of maintained task performance (no vigilance decline was observed) but an increase in subjective fatigue. Furthermore, although we report an expected rise in prestimulus parietal alpha power over prolonged time on task, this was not correlated with the observed rise in subjective state fatigue/mind wandering. Finally, none of our results pointed to theta activity.

## 2 Materials and Methods

### 2.1 Participants

The hypotheses, design and analysis plan were preregistered prior to data collection and can be accessed via the Open Science Framework (https://osf.io/y2vgc/). A total of 41 healthy adults aged between 18 and 87 years old were recruited from the University of Glasgow subject pool and the local area and were given monetary compensation for their time. The study was approved by the University of Glasgow College of Science and Engineering Ethics committee (Approval Number: 300210156). Written consent was obtained from all participants. Participants were balanced for gender and were asked to report any existing medical conditions, eye‐sight correction and medications, which might impact their performance. Seven participants were excluded due to excessive noise and artefact in the EEG signal. The final sample consisted of 34 participants (*F* = 16) split into two groups based on age: young (*n* = 18, *F* = 9, mean age = 22.61, SD = 1.85, range = 20–26) and older adults (*n* = 16, *F* = 7, mean age = 66.50, SD = 8.45 years, range = 55–87). Two participants were left‐handed, one was a smoker and all participants reported low to moderate caffeine consumption on the day (estimated mean units per day = 1.31, SD = 1.12, range = 0–4), corresponding to the maximum recommended daily dose of 400 mg of caffeine (Mitchell et al. 2014). They also reported an average of 7.34 h of sleep per day (SD = 0.85, range = 6–9). All young participants were enrolled university students and the older group had similar levels of tertiary education (*n* = 6, 37.50%) compared to the UK average for their age group (39.60%) (OECD 2023).

All participants were screened for cognitive difficulties using the Montreal Cognitive Assessment test (MoCA; (Nasreddine et al. 2005)), reflecting scores representative of a healthy population (Borland et al. 2017) in both young (mean score = 28.28, SD = 1.49, range = 26–30) and older adults (mean = 25.81, SD = 2.74, range = 22–30), but with the older group having, on average, lower MoCA scores *t*(32) = 3.21, *p* = 0.004. A short (3 min) computerised visual screening assessment was administered at the beginning of the session to exclude potential visual pathologies. The task was adapted from a similar experiment investigating lateralised visual attention in both young and older groups (Learmonth et al. 2017) and shortened to 32 trials. A Welch's *t* test identified no between‐group differences in target detection within the visual regions where the SART stimuli were to be presented, *t*(32) = 1.45, *p* = 0.16.

### 2.2 Subjective Measures

Changes in state fatigue were assessed by the Visual Analogue Scale for Fatigue (VAS‐F). The VAS‐F measures 18 items across two subscales (fatigue = 13 items and energy = 5 items), with scores of 0 = low fatigue to 100 = high levels of fatigue. It has excellent test–retest reliability of α = 0.93 and α = 0.91 for the two scales, respectively (Lee et al. 1991). As in Hanzal et al. (2024), two items on the scale were replaced with synonyms: ‘worn out’ was changed to ‘drained,’ and ‘bushed’ to ‘run down’ to avoid repetitiveness and dated language. The spontaneous subscale from the mind‐wandering measure (Carriere et al. 2013), comprising 4 items on a 7‐point Likert scale, was administered to measure changes in mind‐wandering during the experiment.

### 2.3 Sustained Attention to Response Task

**FIGURE 1:**
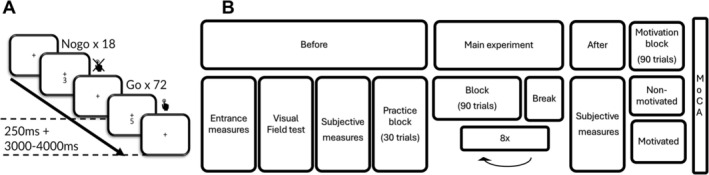
(A) Outline of the SART task. (B) Outline of the experimental procedure. Before the task, participants completed the entrance demographic information, the visual field test and were administered the subjective measure questionnaires assessing state fatigue and mind wandering (VAS‐F, MW‐S). For the main experiment, participants performed 8 blocks of the SART task, allowing tracking of vigilance over time. After completing the experiment, the participants again completed the subjective measure questionnaires (VAS‐F, MW‐S) and proceeded to the final motivational block of SART performance. The MoCA was administered at the end to assess potential presence of cognitive impairments in the sample.

The study used a custom version of the sustained attention to response task (SART) (Robertson et al. 1997) (Figure 1). In each trial, participants were instructed to maintain fixation on a centrally presented cross and attend to a numeric stimulus (0–9) presented at an angular distance of 1° for 250 ms. The fixation reappeared for a variable duration of 3000–4000 ms before progressing to the next number. The stimuli were black on a white background and presented using a 21‐in CRT monitor (Samsung, SyncMaster 1100MB) with a screen resolution of 1024 × 768 pixels and a refresh rate of 100 Hz. Participants were seated 60 cm from the screen, maintaining horizontal eye level with the centre of the display. Participants were instructed to click the left mouse button with their right hand in response to all numbers that appeared (go trials), apart from 3 and 6 (no‐go trials). The participants did not receive any feedback about their individual response times or accuracy. The stimuli were pseudo‐randomised to ensure equal frequency and random distribution throughout the experiment.

### 2.4 Procedure

The experimental procedure is outlined in Figure 1. Participants were provided with an information sheet and gave written, informed consent to take part. They then provided basic demographic information and reported visual deficiencies, as well as any other relevant medical conditions. A 64 Ag/AgCl BrainCap (BrainProducts, Gilching, Germany) was fitted according to the international 10/20 system (American Electroencephalographic Society 1991), including two horizontal electro‐oculographs, and impedance reduced to < 25 KΩ using Signa gel. Participants then completed the VAS‐F and MW‐S and a brief visual field test to map visual acuity. They then underwent a SART practice session of 30 trials and were given general feedback about their accuracy on the practice block. In the main experiment, participants completed eight blocks of the SART (90 trials, 72 go, 18 no‐go trials, random presentation), each lasting 5 min and 20 s, with self‐paced breaks between each block, followed by the VAS‐F and MW‐S to record posttask subjective states. Continuous EEG data were recorded at a 1000‐Hz sampling rate. The first eight blocks of the SART were followed by one further, unannounced, block of 90 trials (Block 9) to manipulate motivational state. Half of the participants were randomised into a motivated group and were instructed to try to maximise their performance on the last block (no guidance was given regarding potential strategies to achieve maximal performance). They were informed that the participant with the best performance during this block would receive an additional bonus of £50. The other half were only informed that the experiment included an additional block and asked to undertake the block with the same instructions as the previous blocks. Finally, the participants were screened for mild cognitive decline using the MoCA and debriefed.

### 2.5 Analyses

#### 2.5.1 Behavioural Analyses

All behavioural analyses were carried out in R (R core team 2020) using the packages ‘tidyverse’ (Wickham et al. 2019), ‘psych’ (Revelle 2023), ‘moments’ (Komsta and Novomestky 2022), ‘readxl’ (Wickham et al. 2019), ‘broom’ (Robinson et al. 2024), ‘ez’ (Lawrence 2016), ‘lmerTest’ (Kuznetsova et al. 2020), ‘lme4’ (Bates et al. 2015) and ‘emmeans’ (Lenth et al. 2024). Further packages used for graphical depiction were as follows: ‘ggpubr’ (Kassambara 2023), ‘viridis’ (Garnier et al. 2023) and ‘Cairo’ (Urbanek and Horner 2023). Performance analyses of the effect of age group and time on the chosen metrics relied on an examination using randomised block‐ and participant‐level modelling. The lmer function of the ‘lme4’ package (Bates et al. 2015) was used to construct the corresponding random mixed effects model and to fit individual participant and block slopes and intercepts, with the lmerTest package (Kuznetsova et al. 2020) to estimate *p* values.

Given the consistent performance differences between age groups, we further examined sensitivity using d‐prime (d′). While not originally included in the preregistration, d′ was chosen as an additional measure of participant sensitivity in the context of possible task strategy differences (Bedi et al. 2024). D′ was computed using the ‘psycho’ package (Makowski 2018), with the function *dprime*, following the formula in (Stanislaw and Todorov 1999) applying an adjustment for extreme values (Hautus 1995). Correct go trials were considered hits, incorrect go trials as misses, correct nogo trials as correct rejections and incorrect nogo trials as false alarms.

#### 2.5.2 Electroencephalography Analyses

Analysis of the electroencephalography (EEG) data was undertaken using the EEGLAB (Delorme and Makeig 2004) and FieldTrip (Oostenveld et al. 2011) toolboxes for Matlab. The continuous data was first detrended to remove drifts introduced by instrumental and physiological noise, alongside various baseline shifts. A Hamming‐windowed FIR filter was then applied within the 2‐ to 45‐Hz frequency range, followed by rereferencing to the average signal. Independent component analysis was then run using ICLabel (Pion‐Tonachini et al. 2019). The following threshold criteria were applied to identify components for automatic rejections: (1) components that had < 0.05 likelihood of brain origin and (2) components that had > 0.8 likelihood to be one of either an ocular artefact, muscle artefact, heart artefact, line noise or channel noise, as per the ICLabel ‘default’ setting. All components labelled as ‘Other’ were visually inspected and rejected if they appeared similar to standard artefact components. This semiautomated correction method led to the rejection of a mean of 25.97 components (of a total of 64 available components per person, SD = 4.25, range = 20–37). A further 1.18 (SD = 1.40, range = 0–3) components were manually rejected in each dataset and 0.24 (SD = 0.55, range = 0–2) preserved from automatic rejection due to incorrect ICLabel classification. The same analytical steps were then performed on the raw datasets with a lower filtering threshold of 0.5–40 Hz, the original ICA weights were reapplied and components removed. Upon inspection of the signal, known noisy electrodes were interpolated (mean per participant = 0.76, SD = 0.96, range 0–4).

Data for analyses were selected based on the following steps:

1. Participants with insufficient neural data as outlined in data preprocessing were not included in behavioural analysis.
2. Trials with trigger information missing due to failure of transition of an event signal were identified and removed.
3. Outlier commission trials were removed. Firstly, block and group mean and standard deviation in reaction time were computed. Blocks were considered separately because of the expected time effect and groups were used because of the effect of cognitive strategy. Then, trials beyond two standard deviations were removed (Kiesel et al. 2008).
4. Participants were also removed if they used either of two incorrect strategies in any of the eight main experimental blocks: (a) responding to all trials (> 80% go stimuli correct and < 20% no‐go stimuli correct) or (b) withdrawing the response for all trials (< 20% go stimuli correct and > 80% no‐go stimuli correct).

Trials thus rejected (based on behaviour) were also identified and removed from the EEG analysis. Further trials were identified for removal based on eye inspection of the signal, detecting ICA‐nonremoved artefact, leading to a rejection of 4.60% of trials (SD = 2.70, range = 0.00%–18.19%).

After initial cleaning, the data was re‐epoched for prestimulus and task‐related analysis. Time‐frequency analysis was performed using a transformation based on multiplication in the frequency domain method as specified in the ft_freqanalysis Fieldtrip function (Oostenveld et al. 2011), and a Hanning taper was applied to the data. The frequency range of interest was defined as 2–40 Hz with a 1/3‐Hz frequency step. The number of fixed cycles per wavelength was set to 6. Cluster‐based permutation testing was employed for comparisons across conditions (Maris and Oostenveld 2007). For all permutation testing, spatial neighbours for each electrode were defined as those being approximately 5 cm distant (Maris and Oostenveld 2007). The maximum possible number of permutations (up to 3000) was undertaken for each test. To investigate whether neural patterns differed across conditions of interest, a permutation test was run on all channels over the whole epoch (−1500 to 1500 ms) applying relative baseline correction and transforming the data to decibels.

Applying the same data cleaning procedure and quality standard in the motivational block led to the selection of different participant data for the motivational group, leading to 33 (older adults = 13). This is explained on a case‐by‐case basis of inclusion of participants based on the overall EEG data quality, which fluctuated between the two parts of the experiment, with a final overlap of 30 participants between both parts. The motivational block data was notably noisier because of a likely higher proportion of agitation and motion artefacts introduced by the motivational manipulation.

## 3 Results

In the current study, we tracked task performance and oscillatory EEG changes in two age groups over time (testing for a time‐on‐task effect) while they performed over 45 min in a SART and explored effects of age differences in strategy, motivation and fatigue on these measures. We also tested for correlations between the behavioural and EEG measures. Our results (i) characterise EEG signatures of the age‐related differences in speed‐accuracy response strategies of older versus younger participants, (ii) identify oscillatory effects of the motivation manipulation implemented in the final block (Block 9) and (iii) test the preregistered predictions regarding EEG‐markers of fatigue (namely, enhanced alpha‐power), which we expected to vary both across age group (greater in the younger adults) and time‐on‐task (increasing across blocks), detailed below.

### 3.1 Age‐Related Differences in Speed‐Accuracy Strategies Over Extended Time‐on‐Task

#### 3.1.1 Performance

**FIGURE 2:**
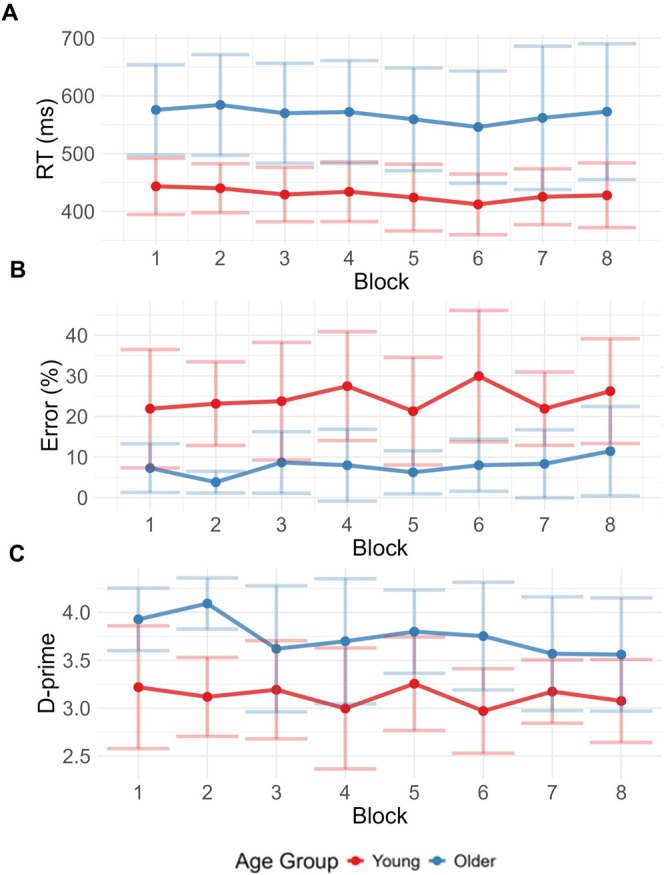
Behavioural performance. (A) Mean reaction time, (B) commission errors and (C) D‐prime for both experimental groups (young in red, older in blue) across time‐on‐task (blocks 1–8).

Across the two groups, the young adults had faster reaction times (mean = 429.45 ms, SD = 50.37 ms, range = 328.88 ms–577.67 ms) but lower accuracy due to elevated commission errors (i.e., a failure to withhold responses; mean = 24.46%, SD = 13.19%, range = 0%–61.11%), while the older group showed a reversed pattern, with slower reaction times (mean = 567.66 ms, SD = 95.18 ms, range = 429.99–923.98 ms) but fewer commission errors (mean error = 7.72%, SD = 7.48%, range = 0%–33.33%) (see Figure 2A,B). Omission errors (missed targets) showed floor effects both in young (0.21%, SD = 1.15%, range = 0%–12.5%) and older adults (mean error = 0.80%, SD = 2.03%, range = 0%–13.89%).

To examine how age‐related speed‐accuracy strategies influenced task performance, we modelled commission errors using a random mixed effects model, with time‐on‐task (Blocks 1–8) and age group (young vs. older adults). There was no effect of time‐on‐task on commission errors [*t*(32) = 0.43, *p* = 0.16], but older adults made fewer errors than the young group [*t*(32) = 15.77, *p* < 0.001], and there was no interaction between age and block [*t*(32) = 0.36, *p* = 0.72] (Figure 2B). An identical model was used to analyse reaction times. There was a small but significant decrease in reaction times across blocks [*t*(32) = 0.01, *p* = 0.049], likely reflecting practice or learning effects. There was also a main effect of age group, with young adults responding faster than the older group [*t*(32) = 0.23, *p* < 0.001], and again no interaction between block and age group [*t*(32) < 0.01, *p* = 0.78] (Figure 2A).

We modelled *d'* using a random mixed effects model with time (blocks 1–8) and age group (young vs. older adults) as predictors. Consistent with the analyses of error and reaction times above, older adults had higher overall sensitivity throughout the experiment [*t*(32) = 5.26, *p* < 0.001], but again there was no main effect of time on *d'* sensitivity [*t*(32) = −0.93, *p* = 0.35]. However, we observed a small interaction between age groups and time [*t*(32) = −2.01, *p* = 0.046]. As shown in Figure 2C, this interaction reflects a slight decrease of sensitivity in the older group over time. However, the older participants maintained substantially higher sensitivity compared to the younger adults throughout the experiment.

#### 3.1.2 Beta‐Activity Changes Reflect Qualitative Differences in Age‐Specific Response Strategy

**FIGURE 3:**
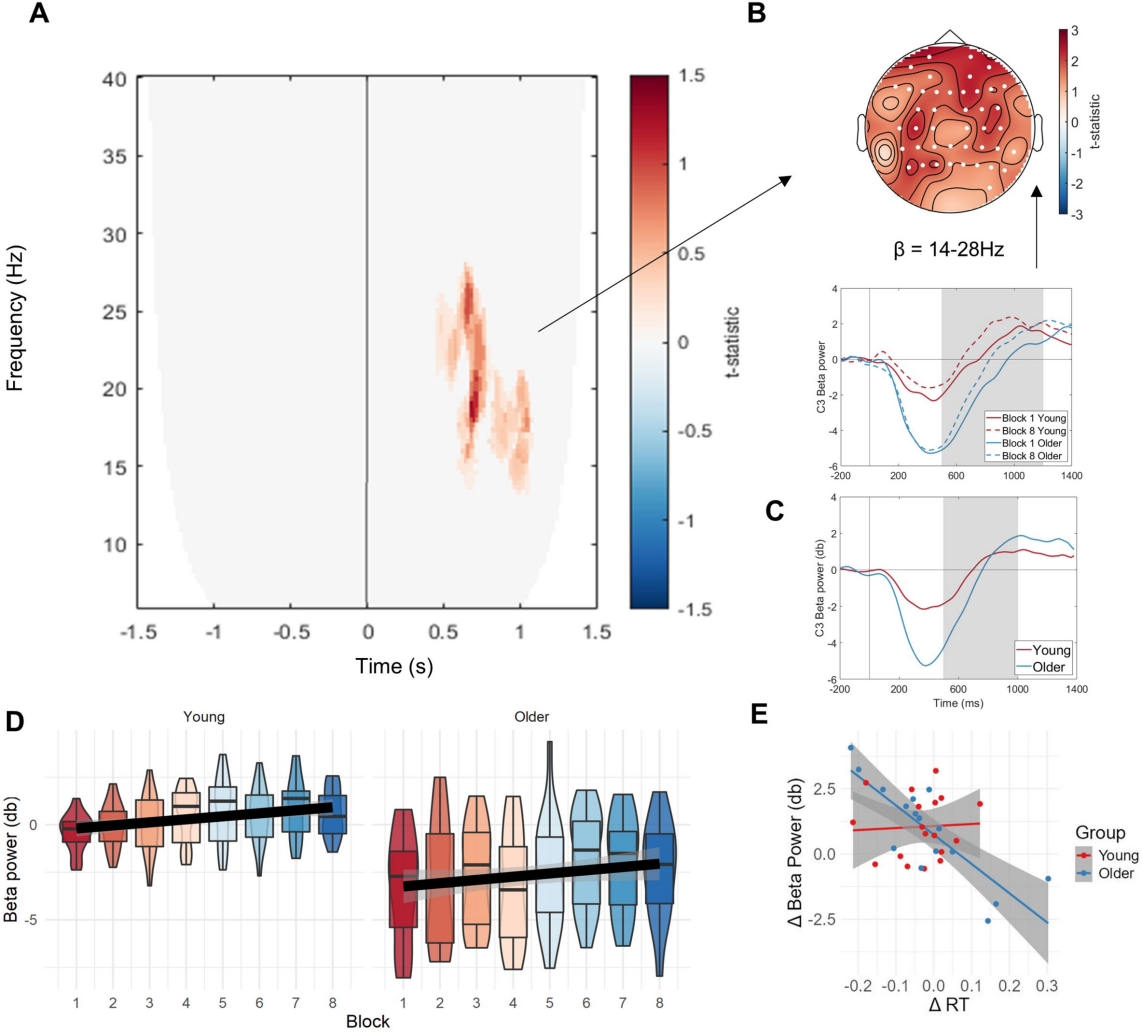
Time‐on‐task related oscillatory changes in the poststimulus period. (A) Results of the cluster‐based permutation test showing a time‐on‐task–related increase in the beta‐band (14–24 Hz) from block 1 to 8 in a later poststimulus window (0.5–1 s). (B) Topography of the time‐on‐task beta‐change, indicating a fronto‐central maxima. The line graph below represents event‐related beta‐changes for blocks 1 and 8 and both age groups illustrating the time‐on‐task increase in beta‐activity of the positive cluster (compare solid vs. dashed lines). (C) Same as the line graph in panel B but taking into account only correct omission trials, hence without contamination by motor execution (data are collapsed across all blocks, see text). (D) Effect of time‐on‐task across all experimental blocks 1–8 per age‐group (young, older), showing an overall positive trend independently of group, but higher beta‐power in the younger participants. (E) Brain‐behaviour links between the change in lower beta power over C3 and reaction times over time‐on‐task for both age groups, showing a negative relationship in older adults.

We first inspected changes over time in task‐related time‐frequency (TF) patterns and, above all, whether these also reflected any age differences. Cluster‐based permutation statistics between blocks 1 and 8 on poststimulus TF‐data identified a positive cluster in the lower beta frequency range (cluster statistic = 4899, *p* < 0.001) occurring in a late poststimulus window (500–1000 ms) (Figure 3A). The cluster is explained by a fronto‐central beta increase over time on task (see map topography in Figure 3B).

To examine beta‐changes across all experimental blocks 1–8, we extracted this beta‐signal (14‐24 Hz: late time window significant in the cluster) from the electrode with the most prominent cluster *t*‐statistic (C3) per block and age group, over time and frequency band (data illustrated in Figure 3D). A random mixed effects model with beta‐power as the to‐be‐predicted variable confirmed its increase across time‐on‐task [*t*(32) = 2.98, *β* = 0.16, *p* = 0.01] and revealed a higher value in the young participant group [*t*(32) = −3.93, *β* = −3.06, *p* < 0.001], but no interaction [*t*(32) = 0.15, *β* = 0.01, *p* = 0.88]. This indicates an overall linear increase of task‐related beta‐power across time‐on‐task, while also showing a stable difference between the age groups.

Inspection of event‐related beta changes (see Figure 3B, line plots below topography) reveals a prominent beta‐desynchronization around manual response onset (mean reaction times were ~ 400–500 ms, see above), followed by a beta‐rebound. The desynchronization was much stronger in amplitude in the older than younger participants, whereas the beta‐rebound showed an earlier latency in the younger compared to the older group and these age differences were comparable across blocks 1 and 8, reflecting the differences in their reaction times (see behavioural results above). Given this dynamic pattern and the fronto‐central topography, we interpret this beta‐signal to reflect differences in motor response strategies between the groups. To further inform this interpretation, we explored to what extent this signal could be driven by the motor response. We therefore reanalysed the beta‐signal, but taking into account correct omission trials only (hence eliminating any contamination by motor execution). Given the low number of omission trials, we averaged the data across all blocks 1–8 (it was not possible to resolve blocks 1 and 8 separately). This analysis revealed the same pattern (see line plots in Figure 3C vs. Figure 3B), including the age‐group differences (*t*(25) = −2.20, *p* = 0.04), suggesting that this beta‐signal is more of a cognitive or motor control nature than linked to motor execution. Based on these findings, we interpret the stronger beta‐desynchronization in the older as compared to the younger group to reflect deployment of more effort towards accurate motor control, while we interpret the shorter latency in beta‐rebound in the young, as compared to the older group, to reflect the speeded response strategy.

#### 3.1.3 Beta‐Behaviour Links

To further consolidate a possible link of the observed oscillatory patterns to age differences in task strategy, we tested to what extent these patterns were also related to behaviour change. Indeed, a model linking change in the pattern to change in reaction times was significant over its most prominent electrode (C3) [*F*(3,30) = 8.84, *p* < 0.001]. While there was no main effect of the beta‐signal (*β* = 0.004, *t* = 0.22, *p* = 0.82), there was (most importantly) a significant interaction between beta signal change and age (*β* = −0.06, *t* = −3.09, *p* = 0.004). An inspection of Figure 3E reveals that the relationship between beta change and reaction time change was driven by the older adult group. Older adults who showed a greater decrease in reaction times showed a greater increase in beta power. Conversely, no relationship was found between error change and task‐related beta [*F*(3, 30) = 0.19, *p* = 0.91] or sensitivity change and task‐related beta change [*F*(3, 30) = 0.94, *p* = 0.43]. The poststimulus beta‐rebound was thus modulated alongside reaction times, giving further indication of poststimulus beta as a likely marker of response strategy differences among the groups.

### 3.2 Effects of the Motivational Manipulation

#### 3.2.1 Performance

The motivational manipulation involved instructing half of the participants, after completing block 8, that they could earn an extra £50 if they outperformed all other participants in the final block (block 9). The remaining participants were simply told that one final block remained, resulting in motivated and nonmotivated subgroups.

We tested the motivational manipulation using a three‐way ANOVA with the factors block (block 8 vs. 9), age group (young, older) and motivational group (nonmotivated, motivated) separately for reaction times, commission errors and sensitivity (*d'*).

**FIGURE 4:**
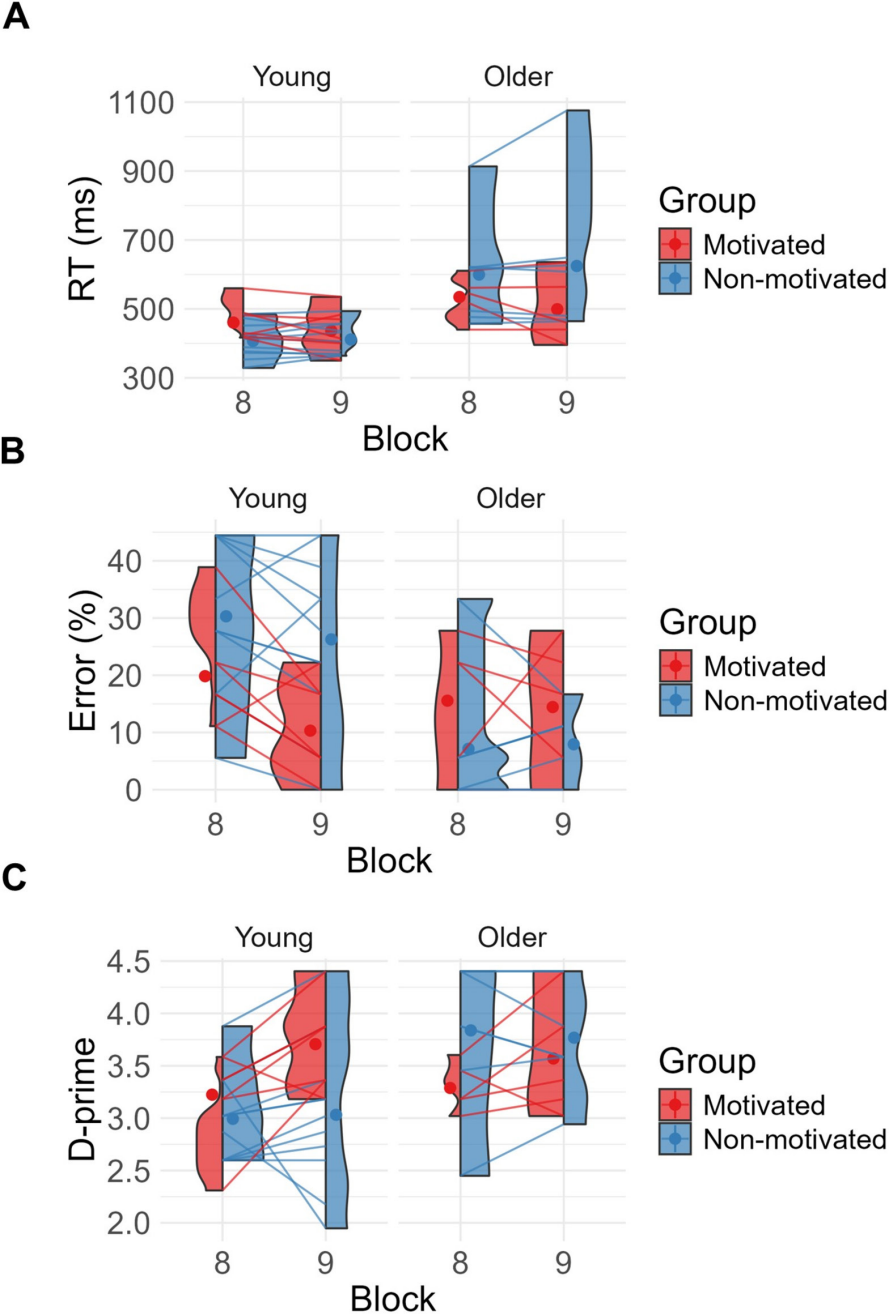
Effects on performance between blocks 8 and 9 following a motivational manipulation in half of the sample across both age groups. (A) Stable reaction time difference between the two age groups and a lack of slowing of reaction times over blocks in the motivated as compared to the unmotivated group, (B) age effects in commission error and (C) in sensitivity measured by d‐prime.

The analysis of the reaction times showed that again overall, the older group had slower reaction times than the young group [*F*(1, 26) = 16.17, *p* < 0.001, *η* ^2^ = 0.42]. There was no effect of experimental block [*F*(1, 26) = 0.37, *p* = 0.55, *η* ^2^ < 0.001] and no effect of motivation group [*F*(1, 26) = 0.18, *p* = 0.68, *η* ^2^ < 0.001], but an interaction between block and motivation was significant with a small to medium effect size [*F*(2, 26) = 7.74, *p* = 0.01, *η* ^2^ = 0.02]. Post hoc analyses were conducted using paired sample *t* tests to further explore the interaction of block and motivation. These revealed that in the nonmotivated group, reaction time increased between blocks 8 and 9 [*t*(17) = −2.47, *p* = 0.02], while remaining stable in the motivated group [*t*(11) = 1.65, *p* = 0.13], hence suggesting that the motivational manipulation affected reaction times. See Figure 4A. No other interaction was significant.

The analysis of the commission errors showed that there was no effect of block [*F*(1, 26) = 1.54, *p* = 0.23, *η* ^2^ = 0.05] and no effect of motivation [*F*(1, 26) = 1.53, *p* = 0.23, *η* ^2^ = 0.04]. The results again showed that the older adults were more accurate than the younger group [*F*(1, 26) = 10.30, *p* = 0.004, *η* ^2^ = 0.24]. Unlike for the reaction times results, there was no interaction between block and motivation, hence suggesting that there was no effect of the motivational manipulation on commission errors. There was a significant interaction between age group and motivation [*F*(2, 26) = 4.77, *p* = 0.02, *η* ^2^ = 0.17], but this interaction was not further explored due to the absence of an interaction with block. This two‐way interaction picks up on a difference between motivated versus nonmotivated young participants independent of block that is not seen in the older participants and is almost certainly reflecting a chance effect. As participants were only allocated to the motivation group randomly after the end of block 8, any signal differences prior to the allocation into the groups would be random variations. See Figure 4B for an illustration of this interaction that is seen throughout all blocks (and is not specific to block 9). No other interaction was significant.

In addition, a similar analysis examined the effect on participant sensitivity. This showed no effect of block *F*(1, 26) = 2.32, *p* = 0.14, *η* ^2^ = 0.02 and no effect of motivation [*F*(1, 26) = 0.002, *p* = 0.96, *η* ^2^ < 0.001] but again confirmed that overall the older adults were more sensitive than the younger group [*F*(1, 26) = 5.22, *p* = 0.03, *η* ^2^ = 0.12]. It further also showed the interaction between age group and motivation [*F*(2, 26) = 7.94, *p* = 0.008, *η* ^2^ = 0.17], which was not further explored for the reasons outlined above. No other interactions were significant; also see Figure 4C.

#### 3.2.2 Task‐Related Beta Is Not Sensitive to the Motivation Manipulation

To test whether the earlier reported time‐on‐task related beta‐signal also co‐varied with the motivational manipulation, this signal was extracted in the electrode with the highest *t*‐statistic (C3) also for the motivational block 9 and separately for the motivated and nonmotivated groups for comparison with block 8. Using a 2 × 2 × 2 ANOVA comparing motivation group (motivated, nonmotivated), age group (younger, older) and block (block 8 vs. motivational block 9), we observed an effect of motivation group [*F*(1, 26) = 4.96, *p* = 0.04, *η* ^2^ = 0.14] showing that the group randomly allocated into the motivational condition had an overall higher beta‐signal. There was no main effect of block [*F*(1, 26) = 0.02, *p* = 0.89, *η* ^2^ < 0.001] or age group [*F*(1, 26) = 3.11, *p* = 0.09, *η* ^2^ = 0.09], nor were there any significant interactions. This indicates that the poststimulus beta‐signal of fronto‐central topography, which is related to response strategy is not amenable to manipulation by motivation.

#### 3.2.3 Motivation‐Induced Beta

**FIGURE 5:**
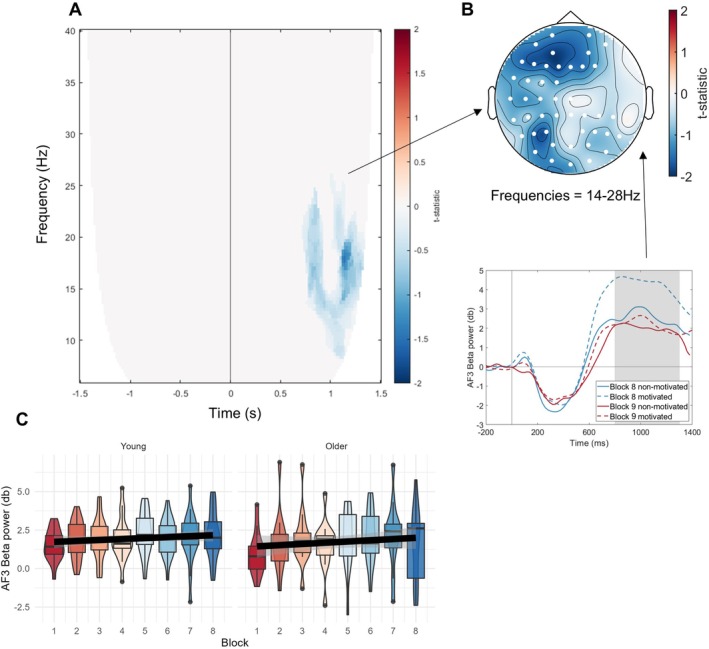
Motivation‐related beta changes. (A) Results of the cluster‐based permutation statistics testing the interaction motivation × block in TF‐space (contrast of the difference signal of block (8 vs. 9) between motivational groups). The results show a late (0.7–1.2 s) effect of motivational manipulation on beta activity (14–28 Hz). (B) Topography and temporal dynamics of the motivation‐related beta‐change in the poststimulus window indicating a left fronto‐parietal decrease in beta‐activity between block 8 and 9 in the highly motivated (dashed lines) but not the nonmotivated participants (solid lines). (C) Evolution of the motivational beta‐signal over time‐on‐task (blocks 1–8) per age group (young, older). The statistics indicate that this beta‐signal does not change over time‐on‐task.

As we did not observe the time‐on‐task related beta‐pattern to be altered by motivational manipulation (as per above), we tested for effects of motivation in poststimulus (task‐related) activity directly through cluster‐based permutation tests. We compared TF differences between blocks 8 and 9 across the two motivational groups (motivated vs. nonmotivated), collapsing across both age groups (interaction of motivation × block in TF‐space). The analysis revealed a broad, late beta‐cluster (Figure 5A), showing a weaker left fronto‐parietal beta‐increase from block 8 to 9 in the motivated relative to the nonmotivated group (statistic = 351.87, *p* = 0.002; see Figure 5B for topography and event‐related beta change).

To examine whether this signal also modulates with time‐on‐task and/or age group, we extracted the motivational beta‐signal (14–28 Hz: in the relevant window) across all experimental blocks 1–8 and both age groups from the electrode with the most prominent cluster *t*‐statistic (AF3) in the test of motivation effects (Figure 5C). A linear model predicting this beta‐power from across blocks and age groups showed no effect of either age [*t*(28) = −0.65, *β* = −0.31, *p* = 0.52] or block [*t*(28) = 1.31, *β* = 0.06, *p* = 0.20], nor their interaction [*t*(28) = 0.23, *β* = 0.02, *p* = 0.82], indicating that these variables had no effect on the motivational beta‐signal and that this signal is hence distinct from the time‐on‐task beta‐effect related to response strategy.

Finally, to confirm the beta‐effects of motivation across all groups/conditions, we run a 2 × 2 × 2 ANOVA with the factors motivation group (motivated, nonmotivated), age group (younger, older) and block (block 8, motivational block 9). The model revealed that the motivated group had higher beta‐signals than the nonmotivated group [*F*(1, 26) = 4.46, *p* = 0.045, *η* ^2^ = 0.12] and that there was a main effect of block [*F*(1, 26) = 21.36, *p* < 0.001, *η* ^2^ = 0.14] with a lower signal in block 9, but no effect of age [*F*(1, 26) = 1.71, *p* = 0.20, *η* ^2^ = 0.05]. The interaction between motivation and block was significant [*F*(2, 26) = 8.55, *p* = 0.007, *η* ^2^ = 0.06] independently of age group (nonsignificant 3‐way interaction motivation group × age group × block: *F*(3, 26) = 2.51, *p* = 0.13). Post hoc analysis exploring the interaction of motivation × block using dependent sample *t* tests revealed that while the signal of the nonmotivated group did not differ between the two blocks [*t*(17) = 1.94, *p* = 0.07], the motivated participants' beta synchronisation decreased [*t*(11) = 4.45, *p* < 0.001]. There was also a significant interaction between age and motivation [*F*(2, 26) = 5.28, *p* = 0.03, *η* ^2^ = 0.14] but this interaction was not further explored, as it almost certainly reflects a chance finding (despite random allocation in age and motivation groups). No other interaction was significant.

In summary, we found a beta oscillation decrease (of fronto‐parietal topography) in the motivated compared to the nonmotivated participants. We also report a levelling out of reaction times in the motivated group (with reaction times in the nonmotivated group continuing to rise between block 8 and 9). Unfortunately, the data proved too sparse to allow for a meaningful brain‐behaviour analysis similar to the previous beta‐signal. Here, motivational group would have to be considered an additional factor to age group, leading to small group sizes when examining a possible relationship between this beta pattern and reaction times.

### 3.3 Effects of Fatigue

We originally preregistered EEG changes with time‐on‐task as possible markers of the subjective measures of fatigue, predicting a link to increases of alpha‐power over time. Accordingly, the EEG analyses were aimed at identifying brain oscillation markers of time‐on‐task and to relate them to any reported subjective changes in fatigue. However, although we found the expected prestimulus rise in alpha‐power, these were unrelated to any behavioural/subjective measures. We report these results below.

#### 3.3.1 Fatigue and Mind‐Wandering Scores

**FIGURE 6:**
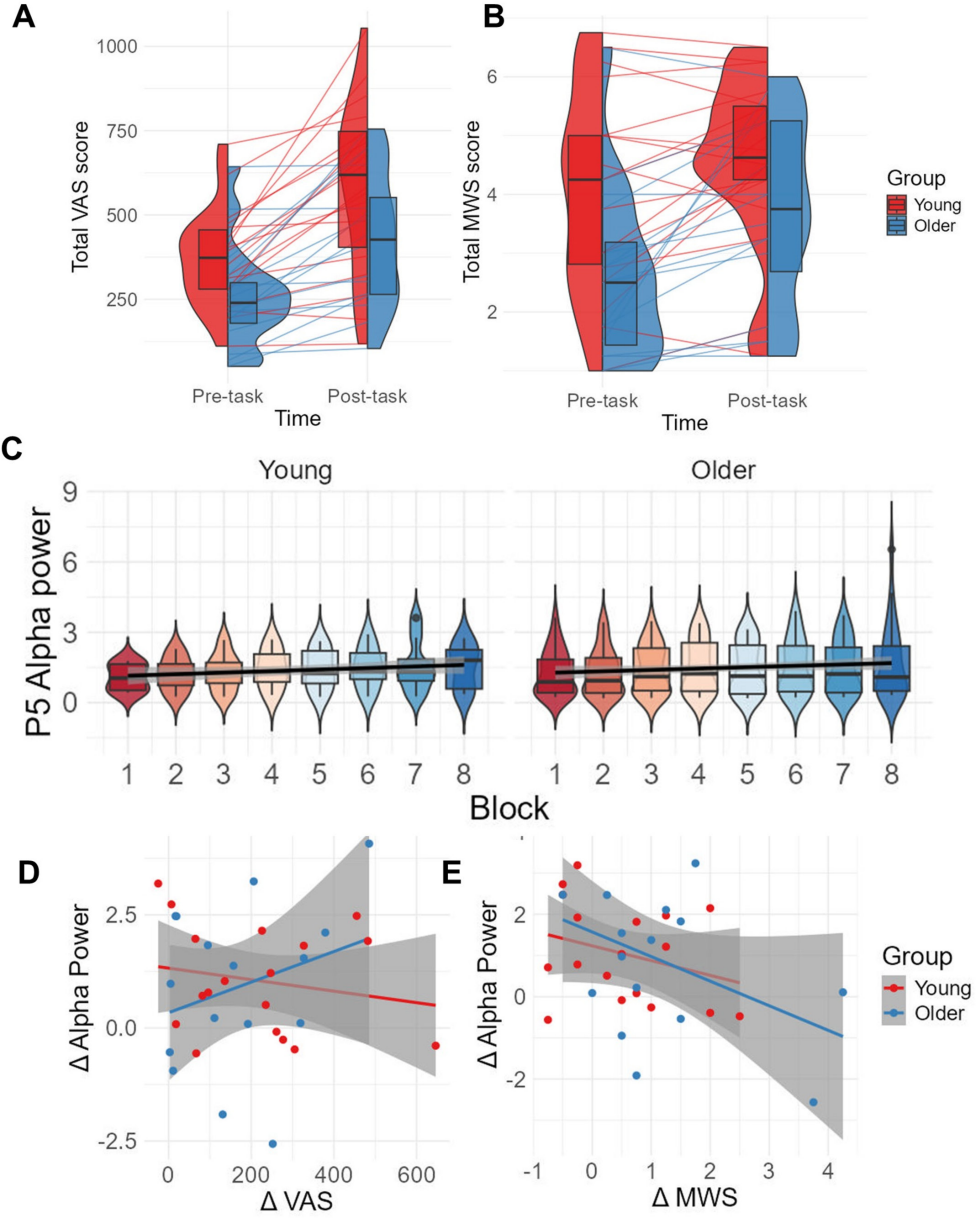
(A and B) Subjective measures. (A) Scores of the visual analogue scale for state fatigue and (B) mind wandering before and after the experiment (pretask, posttask) for each group (young in red and older in blue), with individual participant total scores, as well as overlaid boxplots with overall medians and quartile ranges. (C) Time course of prestimulus alpha‐power over the electrode with strongest cluster‐based differences (P5). The lines illustrate the significant alpha‐increase with time‐on‐task in both age groups. (D and E) Correlations between time‐on‐task effects in EEG and fatigue/mind‐wandering scores. Increase of prestimulus alpha power between blocks 1 and 8 as a function of (D) change in subjective fatigue (VAS) and (E) change in subjective mind wandering (MWS). No significant correlations were observed.

Subjective fatigue levels were assessed before and after task performance (pre block 1 and post block 8). From the beginning, the older group (mean = 427.56, SD = 201.22, range = 104–755) had lower baseline fatigue scores than the young group (mean = 588.89, SD = 257.61, range = 118–1054) [*t*(32) = 2.05, *p* = 0.049, see Figure 6A]. Likewise, from the beginning, older adults had lower mind‐wandering scores (mean = 3.70, SD = 1.61, range = 1.25–6.50) than the younger group (mean = 4.07, SD = 1.71, range = 1–6) [*t*(32) = 2.69, *p* = 0.01, see Figure 6B].

Comparing pretask with posttask subjective scores (see Figure 6A,B), we found that both the young (mean change = 217.06, SD = 182.29, range = −25–646) and older group (mean change = 169.31, SD = 149.58, range = 3–485) showed a rise in their subjective fatigue levels with time‐on‐task. Likewise, both the young (mean change = 0.53, SD = 1.00, range = −0.75–2.50) and the older group (mean change = 1.11, SD = 1.28, range = −0.50–4.25) had an increase in mind‐wandering scores.

In a 2 × 2 mixed ANOVA testing the effect of time (before, after), age group (young, older) and their interaction on the subjective fatigue scores, we found a large effect of time [*F*(1, 32) = 45.75, *p* < 0.001], showing that subjective fatigue scores had increased by the end of the main experiment. There was a small difference between the groups, indicating that the young adults had slightly higher fatigue scores than the older group [*F*(1, 32) = 5.03, *p* = 0.03]. However, there was no interaction between age group and time [*F*(2, 32) = 0.69, *p* = 0.41].

Similarly, in a 2 × 2 mixed ANOVA testing the effect of time (before, after), age group (young, older) and their interaction on mind wandering, there was a large effect of time, explained by higher mind wandering scores by the end of the experiment [*F*(1, 32) = 16.80, *p* < 0.001]. There was again a main effect of age group, with the young adults having higher mind‐wandering scores than the older group [*F*(1, 32) = 5.61, *p* = 0.03]. However, there was no interaction between group and time [*F*(2, 32) = 2.20, *p* = 0.15]. To test for a link between mind‐wandering change and subjective state fatigue change, a linear regression was fitted to the paired observations for each participant, but the model was not significant [*F*(1, 32) = 2.42, *R* ^2^ = 0.04, β < 0.01, *p* = 0.13].

#### 3.3.2 Time‐on‐Task Differences in Prestimulus Alpha Signal

We investigated potential oscillatory markers of time‐on‐task in the prestimulus window. We thought these likely to reflect either correlates of enhanced fatigue/mind wandering or enhanced effort to execute the task at stable performance levels, given the absence of a performance/vigilance decrement over time‐on‐task, despite increased fatigue (from block 1 to 8, see SART results above). To identify the effects of time‐on‐task on oscillatory activity, we compared activity in the prestimulus window (−800 to −300 ms), applying no baseline correction, across a broad spectrum of frequencies (3–40 Hz, including the alpha‐ and beta‐band) between experimental blocks 1 and 8 using cluster‐based permutation statistics (Maris and Oostenveld 2007), in analogy to the analyses above. We then examined whether the markers of time‐on‐task co‐varied with age group.

Examining the effect of time‐on‐task through cluster‐based statistics of the prestimulus activity in experimental blocks 1 versus 8 revealed one positive cluster (cluster statistic = 2599, *p* < 0.001). This indicated a rise in power across time‐on‐task in the alpha frequency band (8–14 Hz) over the majority of channels (maximum effect over P5).

To characterise the alpha‐increase with time‐on‐task over all experimental blocks 1 to 8 and to further test for possible co‐variations with age‐group differences, we extracted the alpha signal (8–14 Hz band) of the prestimulus epoch from the electrode with the highest cluster *t*‐statistic (P5) across all blocks 1–8. Then, we ran an analysis of the influence of the factors time‐on‐task (block 1–8) and age group (young, old) on this signal using a random mixed effects model (with randomised participant‐ and block‐level effects). This was significant across all 8 task blocks [*F*(3, 260) = 4.87, *p* = 0.003], showing no effect of age [*β* = −0.14, *p* = 0.623], but an effect of block [*β* = 0.09, *p* = 0.028] with no interaction [*β* = −0.06, *p* = 0.322], indicating an overall linear increase across time in parietal prestimulus alpha‐power, see Figure 6C.

#### 3.3.3 No Links of Alpha Patterns to Fatigue Measures, Motivation or Performance

To detect links between subjective measures, namely, measures of state fatigue as well as mind wandering and the alpha‐marker of time‐on‐task, we built multiple linear regression models predicting subjective measures by neural signals with the addition of the effect of age and their interaction. Single electrodes from previous permutation tests (see frequency bands/electrodes identified in the above EEG analyses) were extracted and changes in signal were compared to the detected changes in the subjective measures.

The model testing state fatigue (VAS) changes from block 1 to 8 was not significant for its relationship to the corresponding prestimulus alpha‐changes (electrode P5) [*F*(3,30) = 0.27, *p* = 0.605], see Figure 6D. Likewise, no effect was found for the relationship between mind wandering increases from block 1 to 8 and the corresponding prestimulus alpha change [*F*(3, 30) = 2.93, *p* = 0.097], see Figure 6E. An additional correlation analysis on changes in fatigue and mind‐wandering scores versus task‐related beta‐power over time (electrode C3, also showing a time‐on‐task effect, see Figure 3D) likewise revealed no correlations to any of these measures (fatigue: [*F*(3,30) = 0.78, *p* = 0.52], mind wandering: [*F*(3, 30) = 2.43, *p* = 0.09]).

To test whether the detected time‐on‐task rise in the prestimulus alpha power was affected by motivation, we conducted a 2 × 2 × 2 ANOVA. The ANOVA was undertaken with the factors motivation group (motivated, nonmotivated), age group (young, older) and block (block 8, motivational block 9). This revealed no main effects of motivation on this alpha‐signal [*F*(1, 26) = *0*.*723*, *p* = 0.*403*, *η* ^2^ = 0.03], along with no effect of age [*F*(1, 26) = 1.22, *p* = 0.*280*, *η* ^2^ = 0.04], block [*F*(1, 26) = *0*.*008*, *p* = 0.*931*, *η* ^2^ < 0.001] or interactions.

We finally tested whether the detected prestimulus alpha time‐on‐task rise relates to performance. A model linking change in the pattern to change in reaction times over its most prominent electrode (P5) was not significant [*F*(3,30) = 1.09, *p* = 0.370]. Furthermore, no relationship was found between error change and prestimulus alpha change [*F*(3, 30) = 1.31, *p* = 0.29] or sensitivity change and prestimulus alpha change [*F*(3, 30) = 1.19, *p* = 0.333]. We thus conclude that despite our predictions, the observed changes in oscillatory patterns cannot be related to fatigue or mind wandering in our present sample. The parietal prestimulus alpha‐signal thus only showed a rise over time without any links to subjective report, motivational manipulation or performance.

## 4 Discussion

This study sought to identify neural patterns underlying age differences in vigilance, and the influence of strategy, motivation and fatigue, during sustained attention. Our results provide new evidence for two distinct oscillatory patterns associated with an age‐specific difference in response strategy (poststimulus fronto‐central beta) and motivation per se (poststimulus fronto‐parietal beta). Contrary to our expectations, while the reported prestimulus alpha power rise mirrored the subjective rise in fatigue and mind wandering across time and age group, we failed to find a significant correlation between these measures.

### 4.1 Poststimulus, Time‐on‐Task Modulated Beta‐Oscillation Relate to Age‐Specific Response Strategy

We observed a time‐on‐task rise in poststimulus fronto‐central beta synchronisation unaffected by motivational manipulation. This pattern followed the structure of a classic postmotor beta desynchronization and rebound, that is, the neural response related to preparation and/or execution of a motor response (Heinrichs‐Graham et al. 2017; Parkes et al. 2006). In the present study, this beta desynchronisation was much stronger in the older compared to the younger group, whereas the beta‐rebound latency was earlier in the younger group. Given this dynamic pattern, and the fronto‐central topography, we interpret this beta signal to reflect age differences in motor response strategy. More specifically, we think that these age (strategy) differences are of a cognitive or motor control (rather than motor execution) nature because we also found the same pattern for omission trials (which lacked a motor execution aspect). Our thinking is further underpinned by the significant relationship we found between this beta signal change and age: older adults who showed a greater decrease in reaction time over time‐on‐task also showed a greater increase in beta power. This pattern was absent in the younger group. Overall, these analyses suggest that, across age groups, there are qualitative differences in response strategy (accurate vs. fast) that are reflected in a beta signal of fronto‐central topography: We interpret the stronger beta desynchronization in the older, as compared to the younger group, to reflect a deployment of greater effort towards accurate motor control, with the shorter latency in the beta rebound in the younger group to reflect the speeded response strategy. These results further emphasise and document contrasts in age‐specific response strategies reported previously (Dang et al. 2018; Lara et al. 2014; Reteig et al. 2019; Statsenko et al. 2020; Vallesi et al. 2021), but more importantly, we now demonstrate that these qualitative strategic differences are underpinned by distinct beta oscillations (see (Xifra‐Porxas et al. 2019) for similar findings on grip strength).

### 4.2 Poststimulus ‘Motivational’ Beta Oscillation

In addition to the fronto‐central beta‐signature, we found another beta oscillation of fronto‐parietal distribution, which was independent of age group but instead modulated by motivation per se. This signal was stable over time‐on task, yet we found it to show a larger beta‐decrease from block 8 to 9 in the motivated, relative to the nonmotivated, participants. Related to this, reaction times levelled out in the motivated group (from block 8 to 9), while reaction times in the nonmotivated group increased. Our findings thus show that the motivation manipulation was effective, similar to the results of Reteig et al. (2019) who showed a temporary increase in SART performance after an unexpected motivational manipulation. Additionally, the slowing of reaction times in the nonmotivated group coincides with Reteig's findings, reflecting possible disengagement from the task (see also Arnau et al. 2021). We now show this behavioural motivational effect as well as a beta decrease, further adding to the findings of Reteig and colleagues and their detection of decrease in variability of theta response. Beta‐oscillatory changes have previously been linked to motivational manipulations (Wilhelm et al. 2022) and are reactive to changes of internal state (Nickel et al. 2020), although there have been no previous reports on attentional motivation manipulations in human participants.

Our data best reflect the findings and interpretations of Stoll et al. (2016) who examined modulations of frontal beta in monkeys around spontaneous pauses in work. They found that after pauses, the beta power modulation would reset, and the cognitive control effect (task performance) was maintained. We report this signal resetting and maintenance of performance not for pauses in work, but instead for our motivation manipulation. In fact, in line with our data, Stoll et al. (2016) propose that frontal beta oscillations reflect *multiple* factors contributing to the regulation of cognitive control and that motivation parameters can act as modulators of cognitive control.

### 4.3 No ‘Fatigue/Mind‐Wandering’–Related Alpha Oscillation

The task also elicited a distinct linear increase in prestimulus alpha‐power over time, reflecting other research documenting time‐related changes in EEG signal arising from experimental manipulations (Jacquet et al. 2021; G. Li et al. 2020; Tian et al. 2018) and adding to the existing body of literature highlighting detectable changes in alpha oscillations in relation to demanding tasks over time (Benwell et al. 2019; Huycke et al. 2021; Pershin et al. 2023).

With this significant rise in alpha power over time (blocks 1–8), we also note an apparent yet not significant difference in alpha power between the age groups with a greater elevation of the signal in younger compared to the older participants. This would be in line with previous reports finding lower alpha in older participants (Huizeling et al. 2021; Lodder and van Putten 2011; Vysata et al. 2012). In a similar vein, the reported fatigue/mind‐wandering scores were not only elevated over time, but also higher in the younger compared to the older participants. Unexpectedly though, we failed to link these parietal alpha‐power increases to the observed general (and age specific (greater fatigue/mind wandering in the younger participants)) rises in subjective fatigue and mind wandering when testing correlations across individual measures/reports per participants.

It is possible that our subjective measures were not sensitive enough to pick up on correlations with prestimulus alpha. Further work may find better instruments to capture subjective fatigue or the rise in prestimulus alpha may turn out to be unrelated to fatigue.

### 4.4 Limitations and Future Work

Our study reproduced typical age‐related findings in the younger over the older age group, including higher baseline mind wandering and lower reaction times (Diede et al. 2022; Fountain‐Zaragoza et al. 2018; Learmonth et al. 2017), as well as strategy differences (Vallesi et al. 2021) prioritising speed over accuracy (Lara et al. 2014).

What was surprising was the absence of a decisive vigilance decrement, contrary to our hypothesis and in contrast to other work (Gartenberg et al. 2018; Kaufman et al. 2016; Pershin et al. 2023; Reteig et al. 2019; Walker and Trick 2018). The speeding up of reaction times when taking participant‐level randomised intercepts into account replicates our own earlier behavioural findings (Hanzal et al. 2024) with a small effect of time‐on‐task on speed (blocks 1–8). Because participant accuracy did not show a corresponding decline, this result cannot be interpreted as a vigilance decrement. Having consulted previous work (Reteig et al. 2019; Staub et al. 2014) where a decrement occurred at 20–35 min that continued being present in the following hour, we too expected a decline in our study, where the actual task took 45 min, and think that a longer experimental duration would have led to an eventual lapse of this maintained performance (Martínez‐Pérez et al. 2023).

Unexpectedly, although the significant alpha rise over time was mirrored by a significant rise in subjective fatigue and mind wandering, and rises in these measures were also nominally mirrored in age differences (elevated alpha and tiredness/mind wandering in the younger than older participants), we did not find a correlation between these alpha rises and the reported increases in fatigue and mind wandering. It is possible that the subjective fatigue and mind‐wandering scales we used lacked sensitivity specific to the task. Further work is needed to elicit more robust participant responses, possibly through the use of more frequent subjective probes (Weinstein 2018).

In addition, although we found a correlation between the beta signal and the reaction time decreases in the older adults and interpret this stronger beta‐desynchronization in the older group to reflect a deployment of greater effort towards accurate motor control, and the shorter latency in the beta‐rebound in the younger group to reflect the speeded response strategy, it has to be noted that there was no such explicit correlation for the younger group. We also did not set out to measure strategy explicitly as, per preregistration, the main focus of this study was on the EEG correlates of induced fatigue. We did nonetheless, in another behavioural study, force (through titration) both young and older adults to prioritise accuracy over reaction times also in a SART task (Hanzal et al. 2025). Here, had we recorded EEG, we would expect the task‐related beta desynchronization to be the same for both age groups.

Finally, we have to concede that the motivation manipulation (from block 8 to 9) was underpowered. This made it impossible to meaningfully correlate the EEG motivation induced signals with the behavioural measures. The same applies to the 3‐way interactions we describe for some of the behavioural analyses. The original power calculation was for a 2 × 2 design.

## 5 Conclusion

We report that undergoing 45 min of a SART induced subjectively elevated fatigue and mind‐wandering scores alongside a prestimulus parietal alpha power rise. Poststimulus activity revealed two distinguishable beta signatures: a fronto‐central topography as a marker of behavioural strategy and a fronto‐parietal distribution modulated by motivation. We suggest that these two signals reflect a motivational cognitive control mechanism behind resetting a performance decrement. Unexpectedly, although the rises in prestimulus alpha oscillation mirrored the subjective fatigue and mind‐wandering rises that occurred over time, these measures were not correlated, so this signal warrants further investigation.

## Author Contributions

**Simon Hanzal:** conceptualization, formal analysis, investigation, methodology, visualization, writing – original draft, writing – review and editing. **Gemma Learmonth:** conceptualization, methodology, supervision, writing – review and editing. **Gregor Thut:** conceptualization, supervision, writing – review and editing. **Monika Harvey:** conceptualization, supervision, writing – review and editing.

## Funding

This study was supported by the Economic and Social Research Council (Grant ES/P000681/1) and the Wellcome Trust (Grant 209209/Z/17/Z). None of the sources were involved in study design, analysis or preparation of the manuscript.

## Conflicts of Interest

The authors declare no conflicts of interest.

## Data Availability

All demographic and behavioural data files are available from the osf.io database (10.17605/OSF.IO/Y2VGC). Neural data files (EEG) are available upon request.
